# Farm Animal Welfare: Consumers’ Perception Toward Different Breeds of Animals in Italy

**DOI:** 10.3390/ani15233406

**Published:** 2025-11-25

**Authors:** Mariavittoria Perrone, Chiara Mazzocchi, Nicola Palladini, Luciana Bava, Giordano Ruggeri

**Affiliations:** Department of Agricultural and Environmental Science, Production, Territory, Agroenergy, University of Milan, Via Celoria 2, 20133 Milan, Italy

**Keywords:** consumers’ perception, consumers’ interest, farm animal welfare, animal well-being, animal welfare economics, perception of animal welfare

## Abstract

Farm animal welfare has become a key focus in contemporary discussions on ethical food production and sustainable agriculture. Public discourse and evolving consumer attitudes reflect a growing and increasingly sophisticated awareness of animal welfare issues. This study investigates how Italian consumers evaluate the welfare of different farm animals and how such evaluations are influenced by socio-demographic factors, personal attitudes, and the level of knowledge in livestock production systems. Using survey data from 391 respondents and applying regression analysis, the study highlights significant variation in perceived welfare across species. In general, attitudinal variables are the most consistent and significant predictors of perceived animal welfare across all animal categories. Moreover, respondents with professional experience in livestock production tend to view the welfare of cattle and swine more favorably. These findings offer valuable insights for policymakers and stakeholders aiming to align animal welfare standards with public expectations and to promote more informed, ethical consumer choices.

## Simple Summary

This study investigates Italian consumers’ perceptions of farm animal welfare and the factors shaping these assessments. Using survey data from 391 respondents and ordered logit regression analysis, the research identifies significant differences in perceived welfare among species. Attitudinal factors consistently emerge as the strongest predictors of welfare evaluations, while respondents with professional experience in livestock production tend to view the welfare of cattle and swine more positively. The findings enhance understanding of public attitudes toward animal welfare and provide valuable insights for policymakers and stakeholders seeking to align welfare standards with societal expectations and foster more ethical consumer behavior.

## 1. Introduction

Farm animal welfare (FAW) has become an increasingly central topic in discussions on ethical food production and sustainable agriculture [1]. Public discourse and consumer attitudes toward FAW reflect a complex and evolving dynamic, characterized by increasing ethical concern. This is particularly evident in Western societies, where ethical consumerism and growing attention to environmental and social sustainability have amplified calls for more humane animal treatment [2,3,4]. Emotional factors such as sensitivity toward animals, empathy, and ethical value-based beliefs could influence consumer decisions to purchase food products with animal welfare attributes [5,6]. Importantly, perceptions are not uniform across species: cultural norms, intended animal use, and perceived emotional closeness to humans contribute to animal-specific differences in welfare evaluations [7]. From an ethical standpoint, the role of emotional and affective cognition in animals should also be taken into account. Farm animals demonstrate complex social cognitive abilities and nuanced emotional responsiveness, which critically shape their perception of environmental stimuli and mediate the nature and quality of their social interactions [8,9,10].

Although some studies have investigated consumer attitudes toward specific types of farm animals, few have provided a comparative view within a single national context. Moreover, the depth of consumers’ understanding of FAW is frequently limited, because people’s actual knowledge of farming practices, especially in intensive systems, is generally poor [7,11]. The integration of attitudinal and knowledge-based variables into empirical assessments of FAW perception remains underexplored.

This study addresses this gap by examining how Italian consumers evaluate the welfare of different farm animals and how such evaluations are influenced by socio-demographic factors, personal attitudes, and the level of knowledge in livestock system productions. In doing so, the research contributes to a more conceptually robust and scientifically grounded understanding of FAW perception. To our knowledge, this is the first study in Italy to provide a comparative analysis of public perceptions of animal welfare across multiple livestock species, thereby offering novel empirical evidence and a more theoretically and methodologically grounded understanding of consumer perspectives on farm animal welfare.

The study addresses the following research questions:

1. Which factors exert the strongest influence on consumers’ perceptions of FAW?
2. How do the drivers of FAW perception vary across different farmed animal species?
3. How does expertise in intensive animal farming impact perceptions of FAW?

## 2. Consumers’ Perception of FAW

Consumers’ perceptions toward FAW are shaped by a complex interplay of cognitive, affective, and socio-demographic factors [12]. Few studies have focused on understanding different consumers’ perceptions of FAW among animals [12,13]. Sinclair et al. [9] investigated the level of importance attributed to the welfare of animals across countries, including companion animals, i.e., dogs, several farmed animals and wild animals. Their findings show that the importance of animal welfare is acknowledged worldwide, and perceptions vary depending on the animals’ utility. Sullivan et al. [13] focused their research on investigating animal science students’ perceptions in the US, considering FAW across different animal categories. Their survey findings indicate that although most respondents recognize the importance of animal welfare across all species, notable perceptual differences were observed between animal categories concerning specific welfare requirements [13].

Other scholars showed that animals raised for meat, such as pigs and beef cattle, tend to be perceived differently than dairy cattle or laying hens, likely due to varying expectations and emotional associations [14]. Furthermore, extensively reared animals, particularly cattle, were consistently perceived as enjoying higher welfare conditions, independent of the actual husbandry system [15]. In investigating the significance of animal welfare among meat eaters, it has been found that individuals more committed to meat consumption tend to place less importance on animal welfare [16].

Collectively, this literature suggests that consumer perceptions of farm animal welfare are shaped not only by consideration of the animals’ intrinsic needs but also by the broader social roles and symbolic meanings attributed to different species. For this reason, in consumer perception studies, farm animal welfare should be interpreted as a multidimensional construct, reflecting a plurality of values and cultural interpretations that may diverge from the scientific definition of animal welfare.

At the same time, current regulatory and policy frameworks often approach FAW from an anthropocentric perspective, emphasizing its relevance to food safety, consumer confidence, or the economic viability of the agri-food sector. The European Union has progressively formalized animal welfare standards through directives such as Council Directive 98/58/EC and subsequent updates focusing on transport, stunning, and slaughter practices. These regulatory instruments have evolved within broader political strategies such as the “Farm to Fork” initiative and the European Green Deal, which aim to transition toward more sustainable food systems. However, the long-term political viability of the Green Deal is increasingly contested, with mounting resistance from several EU member states, raising questions about the stability of these commitments.

While FAW is often implicitly considered a fundamental component of sustainable agriculture, its absence from the United Nations Sustainable Development Goals (SDGs) reveals a notable gap in the global sustainability framework. SDG 12, which emphasizes responsible production and consumption, could be considered aligned with the ethical dimensions associated with the purchase of animal welfare-certified products [17] but not directly with FAW. This omission is especially striking given the interdependence between animal welfare, environmental sustainability, and public health. Poor animal welfare practices can lead to increased greenhouse gas emissions, resource inefficiency, and the propagation of zoonotic diseases, all of which undermine environmental and human health goals [18]. Furthermore, integrating FAW into sustainability agendas aligns with the One Health approach, which recognizes the interconnected health of humans, animals, and ecosystems [19]. The lack of explicit recognition of FAW within the SDGs may limit the effectiveness of sustainability initiatives and overlook the ethical imperative to ensure the well-being of animals within agricultural systems. Within the European context, initiatives such as the Common Agricultural Policy (CAP) 2023–2027 and the associated Eco-Scheme (ECO1) have begun to acknowledge FAW in relation to climate and biodiversity concerns. From a welfare-economics perspective, these regulatory considerations illustrate that consumers’ perceptions are embedded within broader institutional and policy frameworks, which influence preferences, shape market demand, and ultimately affect the allocation of resources toward FAW-related goods and practices [20].

In contrast, it becomes essential to distinguish between socially constructed perceptions of animal welfare and the ethical and scientific foundations of farm animal welfare. The cognitive capacities of animals, especially their ability to feel pain and other negative emotional states, constitute a crucial basis for evaluating their experiential well-being [8]. Landmark developments, such as the New York Declaration on Animal Consciousness (2023), endorsed by leading neuroscientists and ethologists, affirm that many animals possess the capacity to experience suffering, emotions, agency, and even elements of conscious awareness. Farm animals have rich social cognition and emotional responsiveness, influencing how they perceive their environment and interact [8,9,10]. Consumers, however, often associate animal welfare with broader concepts of fairness, compassion, and naturalness, rather than with formal welfare standards or physiological indicators [7,21]. Consequently, their judgments about animal welfare are influenced by cultural norms, media exposure, and personal identity [22], with empathy and personal values playing a critical role: a greater empathy is often linked to a stronger concern for animal welfare [23].

The literature further indicates that consumers tend to place greater value on FAW when it is supported by regulatory or legal frameworks [4,12,13]. Enhanced FAW standards are also more likely to be acknowledged and trusted by consumers when embedded within established quality assurance or sustainability schemes [4]. These findings highlight that consumer assessments of FAW are not based solely on technical or scientific criteria but are strongly mediated by institutional trust, regulatory legitimacy, and the broader context in which welfare assurances are communicated. Consequently, integrating consumers’ empathy and ethical concerns into the agri-food sector is essential, as affective and attitudinal factors play a critical role in shaping perceptions of FAW.

Another important aspect is that consumers often perceive intensive farming practices as closely linked to negative environmental impacts [24]. Some studies suggest that livestock farming frequently provokes greater concern for environmental impacts than for ethical treatment [11,25]. In contrast, Ammann et al. and Heng et al. show that even when environmental concerns are acknowledged, animal welfare remains a more significant priority for consumers [26,27].

The literature, therefore, presents a nuanced perspective, highlighting the complex and evolving nature of public discourse surrounding FAW. These contrasting findings suggest that consumer attitudes toward environmental issues and ethical considerations are closely intertwined, underscoring the importance of accounting for both when examining perceptions of FAW. Building on these insights, sustainability concerns from a perspective of consumer behavior were addressed in the analysis, as there may be a relationship between the consumers’ opinions as buyers and their perceptions of animal welfare.

In addition, scholars highlight that knowledge about farming practices can significantly influence perceptions of animal well-being [21]. Knowledge represents the informational foundation of consumer perceptions and can function as either a moderator or mediator in the relationship between consumer understanding and behavioral intentions [28]. According to Liu et al. [29], professional experience or educational background in the livestock sector may shape respondents’ perceptions. For example, previous research indicates that cattle farming is often perceived as ethically and environmentally problematic, particularly among individuals lacking professional expertise in the livestock sector [29]. Other studies suggest that direct involvement or expertise in livestock systems can influence attitudes toward welfare by providing practical insights that may either reinforce or challenge preconceived consumer beliefs [30]. These findings suggest that consumers’ attitude towards FAW is likely shaped by subjective interpretation and affective responses, which may differ from expert assessment [21]. However, the influence of professional knowledge on perceptions of intensive livestock systems across different farm animals remains largely unexplored, and this paper seeks to address this gap.

## 3. Materials and Methods

### 3.1. Survey Description and Data Collection

The survey, administered in Italian, consisted of a structured questionnaire focused on consumers’ attitudes and perceptions about FAW. Data were collected through an online questionnaire administered on the Qualtrics platform, using a convenience sampling approach. Participants were recruited by means of a snowball sampling strategy initiated via social media platforms (Instagram, Facebook, LinkedIn), by using a link to the questionnaire introduced by a brief invitation message encouraging participation in the survey. The link to the questionnaire, together with an invitation to participate, was posted only once on webpages related to the agri-food sector. The introduction text was: “The link leads to a short questionnaire related to a study on consumer perceptions of the use of sustainable management practices on livestock farms in Lombardy, taking into account consumers’ knowledge of milk production techniques. The initiative is part of the GO-PEI Vision project, funded by the Lombardy Region and conducted by DISAA of the University of Milan. We kindly invite you to complete the questionnaire available at the following link. Thank you very much for your cooperation”. The link to the questionnaire was shared on the Instagram page of the Food Service and Retail Sciences bachelor program and on the Alleva-menti website of the University of Milan, dedicated to education and research on sustainable livestock farming, on the Facebook page of Aequos, a cooperative of Italian Ethical Purchasing Groups.

This approach was chosen to ensure the inclusion of a sufficient number of respondents with professional knowledge, enabling subsequent comparative analysis. While the “seeding” stage began with a knowledgeable audience, the snowball effect broadened participation to a wider, non-expert population.

As with any non-probability method, snowball sampling carries risks of selection bias and limits external validity. Nonetheless, it is well suited for exploratory research where probabilistic sampling is impractical, as it facilitates access to dispersed populations and supports the accumulation of relatively large datasets [31,32]. In our study, this strategy allowed us to capture variation in professional familiarity with livestock systems while also achieving a reasonable balance across socio-demographic groups.

For this reason, the results should be interpreted as context-specific insights rather than generalizations to the entire Italian population.

Prior to participation, individuals were presented with a comprehensive overview of the study’s aims and procedures, including assurances of anonymity and confidentiality. Informed consent was obtained electronically, with participants affirming that they were 18 years of age or older.

The survey is structured as follows: the first section provides information regarding the answers that would have been collected anonymously and only used for the research goals. At the end of the first section, the question “Do you eat animal-derived products?” is used to identify respondents who do not consume them and exclude them from the analysis. Two vegan respondents were excluded from the final analysis, as their responses were considered outliers. Veganism is typically associated with a distinct and strongly ethical attitude on animal rights, often accompanied by lifestyle choices that differ markedly from those of the general population [33]. As a result, the perspectives of vegan respondents may not be directly comparable to those of non-vegan participants. Whereas the latter are more likely to express views aligned with prevailing societal norms and culturally embedded practices regarding the human–animal relationship, vegans tend to represent a distinct minority perspective that challenges these dominant frameworks. Additionally, given the very small size of this subgroup (*n* = 2), it was not feasible to conduct a meaningful separate analysis or assess potential statistical differences between their responses and those of the full sample. Nevertheless, for completeness and transparency, a regression was run including the vegan respondents (see Table A1, Appendix A). This supplementary analysis confirmed that their inclusion did not materially alter the main findings, providing reassurance that the exclusion of these two cases did not bias the overall results.

The second section gathered socio-demographical information about respondents, such as sex, consistently understood as biological sex, age, level of education, income, professional knowledge about livestock system (PK), and diet habits. The third part of the survey focused on evaluating perceptions of farm animal welfare. Participants were asked directly about their perceptions of well-being for different animal breeds on Italian farms. Responses were collected using a 1–5 Likert scale, where 1 indicated the worst condition (“Poor”) and 5 corresponded with the best scenario (“Excellent”).

A total of 518 raw responses were initially collected. Following data cleaning, which involved excluding incomplete records, the sample size has been reduced to 405. Additionally, given the survey’s length, it has been deemed unreasonable for respondents to complete it in less than 4 min. The minimum completion time was estimated at 4 min, based on an estimated time of approximately 10 s per closed-ended item, summed to obtain a rough minimum. Thus, all responses completed in under 250 s (approximately 4 min) have been removed. The final dataset comprises 391 records, representing 75.5% of the original sample. Data collection spanned from July 2023 to May 2024.

### 3.2. Theoretical Model

The Perceived Well-being (DEP) is the dependent variable derived from the question asked for each animal species: “In your opinion, on a scale from 1 to 5, what is the level of animal welfare guaranteed to farmed animals in Italian farms, taking into account management practices, animals’ needs, living conditions, and the socio-environmental context of the farm?” Where 1 corresponds to “very bad” and 5 corresponds to “excellent”.

The following animal categories were selected for inclusion: dairy and beef cattle, sheep and goats, laying hens and broilers, and swine. These categories represent the most commonly purchased animal-derived products by consumers in Italy. The animals are grouped according to their primary use, i.e., the main purpose for which the animal is raised. Swine, broilers, and beef cattle are classified as meat-producing animals, whereas dairy cattle, laying hens, and sheep/goats are considered producers of dairy products and eggs. Sheep and goats were grouped together because, in Italy, dairy products derived from these two species are often compared and sold together, both in large-scale retail chains and in small local shops. Moreover, the two species share similar farming systems, both in intensive and extensive production contexts. The categorization of animals by their primary use is essential when investigating consumer perceptions of FAW. Meat-producing animals such as swine, broilers, and beef cattle are destined for slaughter, and this fate may influence the perception of their welfare. While it is true that animals raised for milk and egg production, dairy cattle, laying hens, sheep, and goats also eventually end their lives at the slaughterhouse, consumers may experience a greater sense of relief in distinguishing a different primary purpose in the farming of these two animal categories [33].

The set of dependent variables employed in the model is identified by socio-demographic variables (Age, Sex, Education level, Income, and Professional Knowledge) and attitudinal scales referring to Animal Utility (AU), Concern and Empathy towards the Agrifood-system (CEA), and Consumer Behavior (CB). The items for each attitudinal scale were adapted from previously validated literature [1,22,34] and can be found in Table 1. The scales introduce a conceptual approach to find the factors influencing animal well-being based on broader sociological perspectives, social stratification, and existing empirical research. Since attitudes are considered a latent construct and cannot be directly observed, instead of directly measuring them, they must be inferred from overt responses [35]. According to Milfont et al. [35], self-report techniques, such as scales, are the predominant methods used for directly measuring attitudes. Specifically, concerning FAW, given that consumers are ambivalent in their judgment of the agri-food system, the introduction of the CEA scale [1] allows for estimating consumer evaluations of different farming systems across various breeds of animals. Animal farming practices have always raised the ethical issue of exploiting animals for utilitarian purposes. The AU scale [22] reveals the respondents’ acceptance of the use of animals for various motives. Lastly, the respondents’ perception of their degree of influence on society and the environment due to their role as consumers is tested with the CB scale [34].

The survey included a question regarding professional knowledge of intensive farming systems (PK), targeting respondents who engage with such systems as part of their occupation or education. The question was “Do you have in-depth knowledge given by your educational background or professional experience in the field of animal welfare?”. PK was included as a dummy variable in the analysis to examine whether professional familiarity influenced respondents’ assessments. While professional experience may provide greater technical knowledge of intensive farming practices, it does not necessarily guarantee an objective evaluation of farm animal welfare. Professionals in the livestock sector may assess welfare more critically due to their expertise, but they may also be influenced by vested interests or operational constraints, which could bias their evaluations positively or negatively. Moreover, the degree of critical assessment can vary substantially depending on professional role—for example, veterinarians often provide more critical assessments than farmers or other sector professionals [36,37]. Non-professionals, including individuals outside the livestock sector, may offer valuable perspectives that are less constrained by economic or operational interests. Therefore, while PK serves as a measure of technical familiarity, it should be interpreted cautiously, acknowledging that professional respondents may simultaneously bring enhanced knowledge, heightened criticality, and potential biases to their assessments.

The employment of this method represents a novelty in the study of consumers’ perception of FAW. The dependent variable, Perceived Well-being, was regressed for each animal category by using explanatory variables, both socio-demographic and variables derived from the scales: Age, Sex, Education level, Income, PK (socio-demographic variables), AU, CEA, CB (variables derived from the scales).

Before computing the model, the internal reliability of the psychometric scales was checked through Cronbach’s Alpha. Then, we performed the Principal Component Analysis (PCA) on the scales. The answers were based on a five-level Likert scale, where 1 identifies a “strong disagreement” with the related statement while 5 describes a “strong agreement” with the question.

### 3.3. Econometric Model

The ordered logit model is a type of ordinal regression model, designed specifically for dependent variables that are ordinal in nature, with more than two levels. In particular, the survey questions require respondents to choose from “poor,” “fair,” “good,” “very good,” and “excellent.”

Let $y_{i}^{*}$ be an unobserved (latent) continuous variable representing the underlying propensity that determines the observed ordinal outcome y. The relationship between y* and the observed ordinal outcome y is modelled as:

$$y_{i}=j\;\mathrm{if}\;{µ}_{j-1}<y_{i}^{*}\leq {µ}_{j}$$

where $y_{i}$ is the observed ordinal outcome for individual i; *j* denotes the ordinal categories (1, 2, 3, etc.); ${µ}_{j}$ is threshold (cut-point) dividing the real line into intervals corresponding to the observed categories. The latent variable $y_{i}^{*}$ is modelled as a linear function of the predictors $x_{i}$ plus an error term:

$$y_{i}^{*}=x_{i}^{⊤}\beta +\;\varepsilon_{i}$$

where $x_{i}$ is a vector of independent variables for individual i, β is a vector of coefficients to be estimated; and $\varepsilon_{i}$ is the error term, typically assumed to follow a logistic distribution with mean 0 and variance $\frac{\pi^{2}}{3}$.

The probability that the observed outcome $y_{i}$ falls into category *j* is given by:

$${P(y}_{i}=j)=\left({µ}_{j-1}<y_{i}^{*}=j\leq {µ}_{j}\right)=P({µ}_{j-1}<\;x_{i}^{⊤}\beta +\varepsilon_{\;i\;}\leq {µ}_{j})$$

Given the logistic distribution of the error term $\varepsilon_{\;i}$ the probability can be written as:

$${P(y}_{i}=j)=\wedge \left({µ}_{j}-x_{i}^{⊤}\beta \right)=\wedge ({µ}_{j-1}-x_{i}^{⊤}\beta )$$

where ∧ (z) is the cumulative distribution function of the logistic distribution:

$$\wedge \left(z\right)=\frac{1}{1+e^{-z}}$$

Thus, the probability for the ordered outcomes is:

$${P(y}_{i}=1)=\wedge \left({µ}_{1}-x_{i}^{⊤}\beta \right)$$

$${P(y}_{i}=2)=\wedge \left({µ}_{2}-x_{i}^{⊤}\beta \right)-\wedge \left({µ}_{1}-x_{i}^{⊤}\beta \right)$$

$${P(y}_{i}=J)=1-\wedge \left({µ}_{J-1}-x_{i}^{⊤}\beta \right)$$

The model is estimated using maximum likelihood estimation (MLE), with the likelihood function constructed based on the probabilities defined above. Estimation is carried out in Stata 18 using the ologit command, which is specifically designed for ordered logistic regression.

## 4. Results

### 4.1. Descriptive Statistics

The socio-demographic details of the respondents are summarized in Table 2. The sample used in this study demonstrates substantial demographic variation across key variables, including sex, age, education, income, and professional knowledge of intensive livestock system (PK). Specifically, while females constituted a higher proportion of the sample, males were also well represented. The age distribution covered a broad range, with the largest segment aged 50–64 (32%), followed by 35–49 (22%), ensuring representation across multiple life stages. Educational attainment was diverse, with a majority holding either a high school diploma (42%) or a university degree (38%). In terms of income, responses were distributed across brackets, with the most common being EUR 25,000–49,000 annually (30%), while 25% opted not to disclose this information. Notably, 76% of respondents reported no professional knowledge of livestock systems; the remaining 24% reported having expertise in this sector. This indicates that the sample includes a fairly substantial proportion of individuals with professional knowledge, as expected by the sample collection in social media with profiles dedicated to agricultural sector professionals.

All demographic variables exhibited weak statistically significant non-uniform distributions (chi-square tests, all *p* < 0.05), confirming heterogeneity within the sample.

In evaluating the perception of animal well-being, the survey assessed various aspects of consumers’ opinions on animal welfare grouped by breed of animals using a 1 to 5 Likert scale. Table 3 explains data collected concerning the dependent variable, “Perceived Animal Well-being”. Initial analysis reveals varying levels of perceived welfare across bred animals. Dairy cattle received the highest mean perceived well-being score, followed by sheep/goats and beef cattle. The poorest perceived condition is noted for broilers, followed by swine and laying hens raised for egg production. A one-way ANOVA assesses significant differences in perceived well-being across animal categories (*p* < 0.0001).

Tukey’s HSD (Honestly Significant Difference) test (see Table A2, Appendix A) revealed that most pairwise comparisons were statistically significant. In particular, broilers were rated significantly lower than all other animals, while dairy cattle and sheep/goats were rated significantly higher than most others. In other words, people rate the well-being of animals like dairy cows, broilers, and pigs differently, and these differences are unlikely to be due to chance.

### 4.2. Model Results

Table 4 shows the different items included in each attitudinal scale and the scales’ reliability. The internal coherence among the different items of the attitudinal scales has been estimated through the calculation of Cronbach’s Alpha. Cronbach’s alpha helps evaluate whether the items in a scale or test consistently reflect the same concept and, therefore, whether the scale is likely to produce reliable results.

The initial Cronbach’s α value for the CEA construct was 0.521, indicating inadequate reliability. To address this, the third item, identified by the lowest factor loading, was removed, resulting in an improved reliability score of 0.629. Although this value remains below the conventional threshold of 0.7, it is consistent with the reliability reported by Schenk [39] and is considered acceptable for exploratory research. Furthermore, model results indicate that this scale plays a significant role in explaining the dependent variable, highlighting its relevance within the overall model. The remaining constructs demonstrated satisfactory internal consistency, with Cronbach’s α values around 0.7 for AU, and high reliability for CB, which achieved an α of 0.861.

Given the satisfactory internal consistency of the scales employed in this study, Principal Component Analysis (PCA) was performed separately for each of the three scales. In all cases, only the first component exhibited an eigenvalue greater than one; therefore, one component per scale was retained in accordance with Kaiser’s rule. The corresponding PCA results are presented in Table 4. PCA reveals that Consumer Behavior (CB) is the most robust factor, explaining 64.8% of the variance with the highest reliability (Cronbach’s α = 0.861). Animal Utility (AU) and Concern and Empathy towards the Agri-food system (CEA) exhibit acceptable explanatory power, respectively 59.4%, and 73.2% of the total variance explained. Table 5 presents the model results, all of which indicate a good fit according to the Chi-squared test.

The regression analysis revealed that attitudinal variables were the most consistent and significant predictors of perceived animal welfare across all animal categories. All variables included in the model exhibited acceptable levels of multicollinearity (VIF ≤ 5), supporting the robustness and reliability of our results. Some predictors consistently influence perceived animal welfare across all animal categories, although the magnitude and significance of these effects vary, reflecting nuanced consumer perceptions. Concern and Empathy towards the Agrifood-system (CEA) stands out as the most robust and uniformly significant predictor, with the highest coefficients observed for dairy cattle (1.212) and beef cattle (1.178), followed closely by swine (1.145) and broilers (1.124). This may suggest that empathy-driven attitudes toward the agri-food system seem to be influential in shaping positive perceptions of welfare for animals more closely associated with intensive production systems.

Animal Utility (AU) also shows a positive and significant effect through all farm animals, but its influence is more pronounced for beef cattle (0.531) and swine (0.493), two meat-producing animals. In contrast, AU’s effect is weaker for sheep/goats.

Consumer Behaviour (CB) is significant only for dairy cattle and broilers, with negative coefficients (−0.239; −0.256), suggesting that higher scores on the CB scale are associated with lower perceived welfare for these animal categories. Professional Knowledge (PK) significantly increases welfare perceptions for dairy cattle (0.862), beef cattle (0.985), and, to a lesser extent, for swine (0.476), but not for other categories.

In general, socio-demographic characteristics appear to have minimal influence on the dependent variable. Gender shows a significant negative effect only for sheep/goats (−0.588), indicating that women tend to have a lower perception of animal welfare in the case of sheep and goats. Age effects are more scattered, but middle-aged groups (35–64) show higher welfare perceptions for beef cattle, laying hens, and broilers, with the strongest coefficients for broilers (0.956) and beef cattle (0.860) in the 50–64 age group. This could suggest a generational difference in perceptions. Higher education is weakly associated with lower welfare perceptions only for laying hens (−2.938) among university-educated respondents. Thus, compared to other factors, socio-demographic characteristics seem to exert only a marginal effect.

## 5. Discussion

Interesting remarks can be drawn from both the descriptive analysis and the interpretation of the regression models. The results indicate that attitudinal factors could be important in influencing how consumers perceive FAW across various livestock animals. The results suggest a pattern consistent with the findings of Kendall [18], who emphasized the role of broader sociological and emotional factors, such as moral worldviews, in shaping animal-related attitudes.

The regression analysis revealed that among the attitudinal variables examined, CEA emerged as the most robust and consistent predictor of perceived welfare, particularly for animals associated with more intensive production systems, such as dairy cattle, beef cattle, swine, and broilers. Individuals who demonstrate higher levels of empathy for the agri-food system tend to perceive farm animal welfare more positively, among all the animal categories. Interestingly, the influence of CEA was strongest for dairy cattle and beef cattle, which may reflect consumer perceptions of these animals as being raised in less confined or more careful systems. Previous studies have shown that visual imagery and cultural narratives associated with open pasture or small-scale livestock farming can positively influence welfare perceptions [7,15]. Moreover, the result is in line with Ritter et al. [40] showed that, regardless of the farming system (intensive or extensive), dairy cows were perceived as having a higher level of welfare compared to other species. The study also found a strong positive association between emotional responses and welfare perception. In fact, animals that people felt more emotionally connected to, such as cows, were perceived as having better welfare conditions, even when this perception did not necessarily reflect the reality of the production systems. This interpretation is consistent with Vanhonacker et al. [21], who highlight that citizens’ judgments of farm animal welfare are primarily influenced by affective and value-laden attitudes, often resulting in more positive welfare assessments for species with which individuals feel a stronger emotional connection [21].

The slightly lower coefficients for laying hens and sheep/goats may be attributed to the association of these species with a less well-known production system or less empathy towards these animals. Although hens and sheep/goats are also raised intensively, they are likely perceived by our sample as being more closely associated with mixed livestock systems. In the case of hens, they are traditionally seen as classic backyard animals [41], and in the case of sheep/goats, they are generally associated with grazing, pastures and mountains [42]. In this sense, concern and empathy toward the agri-food system may have less influence on the perception of animal welfare for these species compared to their impact on the perception of other animals included in the study.

The observed positive and significant effect of AU across all farm animal species reflects a prevailing utilitarian worldview in which animal welfare is valued to the extent that it serves or enhances human objectives, aligning with welfarist conceptions of ethical animal use [43]. Moreover, the role of AU as a positive predictor of FAW perception, particularly for beef cattle and swine, suggests that acceptance of animals’ instrumental roles within the food system moderates ethical concerns and supports more favorable welfare evaluations. This may be interpreted through the lens of moral disengagement theory [44], whereby individuals justify animal use by focusing on productivity or necessity, thereby reducing the cognitive dissonance inherent in the meat paradox—the conflict between concern for animal suffering and continued meat consumption [45,46].

In addition to attitudinal variables, the analysis reveals that Professional Knowledge (PK) significantly influences welfare perceptions for dairy cattle, beef cattle and swine, but not for other livestock categories. Given that PK is a dichotomic variable, its effect reflects a systematic difference in perceptions between respondents with and without professional knowledge. This pattern may indicate that respondents with professional knowledge have a greater familiarity with FAW, which informs their evaluations. The effect supports the idea that increased knowledge does not universally lead to more critical perspectives. In Italy, the dairy and meat sectors, traditionally dairy cattle and swine, may be viewed more positively by professionals due to both personal experience and established quality standards [47]. On the contrary, professional knowledge may reinforce perceptions of legitimacy or efficacy within specific production systems, especially when individuals identify with the values or practices of those systems [3], revealing a potential conflict of interest between professional alignment and objective assessment of animal welfare. Furthermore, this result may reflect a broader phenomenon described in the literature as the “professional gaze,” where individuals embedded within a system tend to normalize practices and evaluate welfare based on feasibility and standards internal to the system, rather than public expectations [48].

Overall, the results highlight the complexity of consumer attitudes toward FAW and the animal-specific variations in how empathy toward the system, utilitarian thinking, and critical views influence perception. These findings reinforce prior literature suggesting that attitudes are not only strong predictors of ethical food choice but also shape cognitive interpretations of production realities [4,28]. As regards to professional knowledge variable, findings are consistent with previous research indicating that familiarity with agricultural systems can moderate critical attitudes, particularly when such exposure is associated with transparency, regulated practices, or traditional farming models [29,49].

The practical implications of this work relate both to the market and public policies on animal welfare. From a market perspective, a first consideration should be given to FAW consumers’ perception. According to our study, consumers exhibit different perceptions of animal welfare, particularly regarding the possibility and appropriateness of using animals for human purposes, depending on the animal species. From the market and producers’ perspective, this is a result that should lead to communicating animal products differently according to the species type. Greater attention should be given to products derived from chicken and pigs, which still garner feelings of mistrust and low confidence among consumers, specifically in terms of animal welfare. Attention should be focused on the target consumers to aim for, as gender and age influence the perception of FAW and can determine purchasing choices. Indeed, in line with Vanhonacker F. et al. [21], young people and women seem to be more sensitive, thus opening a niche market for communicating sustainable farming practices or emphasizing the quality of farming in terms of animal welfare to these population segments.

Findings regarding public policies underline the importance of reducing the asymmetry of information between producers and consumers. This represents an important market failure in agri-food economics that institutions should address through the establishment of an effective and harmonized regulatory framework, driving incremental enhancements in the sustainability of human food production. In fact, public attitudes toward FAW will impact farm animal policies and legislation, influencing their treatment [30,50,51]. Public attitudes are considered a powerful catalyst for enhancing FAW.

## 6. Conclusions

This work represents the first attempt to study consumers’ perception of animal well-being across different farm animals in a sample of the Italian population, using attitudinal scales. The model seems suitable to study consumers’ perceptions, and some findings happen to be in line with previous research. The strong predictive role of consumer empathy toward the agri-food sector and of critical attitudes toward livestock production systems suggests that public concerns regarding animal farming practices are central to perceptions of farm animal welfare. These findings support the growing consensus in the literature that effective FAW governance must move beyond a reliance on legislation alone and toward more pluralistic institutional approaches. While regulation remains a necessary foundation, particularly in addressing baseline welfare conditions and enforcement, complementary strategies such as voluntary labeling schemes, certification systems, and animal welfare indicators embedded in quality assurance programs can help bridge the gap between production realities and public expectations. While EU legislation, such as Directive 98/58/EC on the protection of animals kept for farming purposes and species-specific directives (e.g., Directive 2008/120/EC for pigs, 1999/74/EC for laying hens), sets minimum welfare standards, research and practice increasingly point to the limits of legislation alone in addressing evolving public expectation [52]. The European Commission’s Farm to Fork Strategy (2020) recognizes this, emphasizing the need for enhanced labeling, transparency, and stakeholder engagement to rebuild trust and stimulate higher-welfare consumption. Voluntary mechanisms such as animal welfare labeling, private certification schemes, and retailer-led standards have emerged as vital tools to complement regulation [53]. In Italy, initiatives such as the Sistema di Qualità Nazionale per il Benessere Animale (SQNBA) and retailer partnerships with third-party certifiers are expanding the availability of certified higher-welfare products, especially in the poultry and dairy sectors. These programs not only provide market incentives but also create a feedback loop between production standards and consumer expectations. In addition, marketing strategies for animal-based products could be more effectively tailored to care-oriented consumers by acknowledging the species-specific differences in perceived animal welfare. By aligning communication efforts with consumers’ ethical concerns and values, particularly regarding the treatment of different farm animal species, producers and retailers may improve their visibility, ultimately influencing purchasing behavior. Furthermore, meaningful stakeholder engagement, including producers, consumers, veterinarians, animal welfare organizations, and policymakers, could be essential not only for enhancing the perceived legitimacy of animal welfare improvements but also for ensuring their practical feasibility. The integration of diverse perspectives can support the development of more context-sensitive and socially accepted welfare standards.

Overall, these findings highlight the need for a multidimensional approach to farm animal welfare (FAW), one that incorporates consumer psychology, species-specific perceptions, and participatory governance. Future research should further explore how public perceptions can be translated into concrete welfare improvements across different production systems, thereby fostering a more ethically aligned and socially sustainable food system.

## 7. Limitations

Despite the valuable insights generated by this study, several limitations should be acknowledged to contextualize the findings and guide future research.

The data were collected through a self-administered online questionnaire, relying on voluntary participation. This sampling approach, while effective for reaching a broad audience, may introduce self-selection bias and limit the representativeness of the sample. Individuals with strong pre-existing views on animal welfare may have been more inclined to participate, potentially skewing the results. Moreover, it was not possible to include respondents following a vegan diet in our analysis. These consumers may have different perceptions of farm animal welfare compared to other individuals, which could have produced particularly interesting results. Future studies could benefit from employing probabilistic sampling methods or incorporating mixed-mode survey designs—such as combining online and face-to-face interviews—to improve representativeness across demographic and geographic groups.

Then, while the study provided detailed insights into how attitudes shape welfare perceptions across different livestock categories, it did not examine consumers’ behavioral intentions, such as their willingness to pay for higher welfare products. Prior research has indicated that attitudes and perceptions do not always translate into concrete consumer behavior, a phenomenon often referred to as the attitude-behavior gap. Further research should investigate how differing perceptions of animal welfare, particularly among species associated with intensive vs. extensive production systems, influence WTP and purchasing choices. This could be achieved by integrating discrete choice experiments or conjoint analysis into the survey framework.

The analytical approach employed allowed for robust identification of predictors but was limited in its ability to capture complex interdependencies among attitudinal constructs. Future work could adopt advanced data analysis techniques, such as structural equation modeling, to better understand the latent structure and interactions between empathy, utilitarian thinking, critical concern, and knowledge. These methods could uncover attitudinal profiles or typologies that inform more targeted policy and communication interventions.

In sum, addressing these limitations in future research will not only enhance the robustness of findings but also contribute to a deeper understanding of how public attitudes toward farm animal welfare are formed, expressed, and acted upon, critical knowledge for designing effective and inclusive governance frameworks.

## Figures and Tables

**Table 1:** Description of the attitudinal scales used in the research.

Variable	Description	Items	Reference
AU—Animal Utility	Refers to the concept of general utilitarianism	As long as animals do not suffer pain, humans should be able to use them for any purpose;	[22]
	Aims to assess the degree to which individuals believe that human needs can overcome the needs of animals	It is acceptable to use animals to test consumer products such as soaps, cosmetics, and household cleaners;	
		Hunting animals for sport is an acceptable form of recreation;	
CEA—Concerns and Empathy towards the Agri-food system	It aims to evaluate concerns and empathy developed by consumers towards the agri-food system	Current labels on animal products make it possible to identify the rearing and welfare conditions of the animals;	[1]
	Each item of this scale examines a different aspect of the agri-food system: trust in farmers, trust in food labelling and economical compensation mechanisms related to the enhancement of farm animal welfare	Living conditions for farm animals in Italy have improved over the past 10 years;	
		Farmers should be compensated economically for the increased costs associated with improving animal welfare;	
CB—Consumer Behaviour	Consumers can have different levels of awareness regarding the outcomes of their behaviour	What each individual consumer purchases largely determine the extent of the problems a nation’s environmental issues;	[34]
	When consumer behaviour is applied to any sustainability issues, it should be defined through the concept of Societal consumer instrumentality awareness (CIA-S)	The efforts of each individual consumer to purchase products with a low impact on the environment contribute significantly to the reduction in pollution;	
	CIA-S reflects consumers’ understanding of how their individual consumption choices can contribute to addressing specific issues or problems	Every single consumer can significantly influence society by purchasing products from socially responsible companies;	
		Every consumer who purchases fair trade products contributes substantially to a more equitable society;	
		The purchasing behaviour of each individual consumer has a great effect on the welfare conditions of workers;	

**Table 2:** Descriptive statistics of the sample population in comparison with national averages from the Italian population (Istat data).

Sex	Frequency	Percentage (%)	Median	National Data (2024) Frequency [38]	Percentage (%)
Male	158	40.4	Female	28,814,832	49%
Female	233	59.6		30,182,369	51%
Age Groups				National Data (2023) Avg [38]	
18–24	75	19.18	35–49	46.6	
25–34	78	19.95			
35–49	86	21.99			
50–64	124	31.71			
over 64	28	7.16			
Education Level				National data (2020) [38]	
Primary School	2	0.51	High School	8,262,985	16%
Middle School	38	9.72		16,733,174	32%
High School	165	42.20		19,037,299	37%
University	148	37.85		7,943,764	15%
Postgraduate/Doctoral	38	9.72			
Income (Gross EUR/year)				National Avg individual income (2023) (EUR/year) [38]	
Below 15 K	34	8.70	Between 16 K and 24 K	21,553	
Between 16 K and 24 K	75	19.18			
Between 25 K and 49 K	116	39.67			
Between 50 K and 69 K	44	11.25			
Above 70 K	25	6.39			
I prefer not to say	97	24.8			
PK—Professional Knowledge					
yes	93	23.79	no		
no	298	76.21			

**Table 3:** Descriptive statistics of the dependent variable (Perceived Animal Well-being) of each regression model, total sample.

Perceived Animal Wellbeing	Obs.	Mean	Std. Dev.	Min	Max
Dairy cattle	391	3.20	1.11	1	5
Beef cattle	391	2.86	1.19	1	5
Swine	391	2.46	1.18	1	5
Laying hens	391	2.53	1.24	1	5
Broilers	391	2.18	1.19	1	5
Sheep and Goats	391	3.17	1.10	1	5

**Table 4:** Results of PCA and Cronbach’s Alpha test for each scale.

Variable	Question	Factor Loading	Eigenvalue	% Variance Explained	Cronbach α
AU—Animal Utility			1.781	0.594	0.654
	As long as animals do not suffer pain, humans should be able to use them for any purpose;	0.622			
	It is acceptable to use animals to test consumer products such as soaps, cosmetics, and household cleaners;	0.571			
	Hunting animals for sport is an acceptable form of recreation;	0.535			
CEA—Concern and Empathy towards the Agri-food system			1.465	0.732	0.629
	Current labels on animal products make it possible to identify the rearing and welfare conditions of the animals;	0.707			
	Living conditions for farm animals in Italy have improved over the past ten years;	0.707			
CB—Consumer Behavior			3.241	0.648	0.861
	What each individual consumer purchases largely determine the extent of the problems a nation’s environmental issues;	0.386			
	The efforts of each individual consumer to purchase products with a low impact on the environment contribute significantly to the reduction in pollution;	0.473			
	Every single consumer can significantly influence society by purchasing products from socially responsible companies;	0.473			
	Every consumer who purchases fair trade products contributes substantially to a more equitable society;	0.453			
	The purchasing behaviour of each individual consumer has a great effect on the welfare conditions of workers;	0.446			

**Table 5:** Results of o-logit regressions for each animal breed (Dairy Cattle, Beef Cattle, Swine, Laying hens, Broilers, Sheep/Goats).

	Dairy Cattle						Beef Cattle						Swine					
Predictor	β	SE	z	*p*	Odds Ratio	95% CI for OR	β	SE	z	*p*	Odds Ratio	95% CI for OR	β	SE	z	*p*	Odds Ratio	95% CI for OR
Sex Female = 1	−0.404 ●	0.229	−1.76	0.078	0.668	0.426–1.047	−0.345	0.223	−1.55	0.121	0.709	0.458–1.096	−0.04	0.224	−0.18	0.860	0.961	0.620–1.490
25–34	0.309	0.338	0.91	0.362	1.361	0.702–2.641	0.287	0.328	0.88	0.382	1.332	0.701–2.534	0.431	0.327	1.32	0.187	1.539	0.811–2.918
35–49	0.259	0.313	0.83	0.407	1.296	0.702–2.392	0.520 ●	0.306	1.7	0.090	1.682	0.923–3.066	0.43	0.3	1.43	0.152	1.537	0.854–2.769
50–64	0.298	0.284	1.05	0.293	1.348	0.773–2.350	0.860 **	0.286	3.01	0.003	2.363	1.349–4.139	0.468 ●	0.285	1.64	0.101	1.597	0.913–2.791
over 64	0.507	0.447	1.13	0.257	1.660	0.691–3.989	0.378	0.420	0.9	0.369	1.459	0.640–3.325	−0.054	0.444	−0.12	0.903	0.947	0.397–2.263
Middle School	−1.682	1.409	−1.19	0.233	0.186	0.012–2.944	0.445	1.192	0.37	0.709	1.56	0.151–16.128	−0.64	1.175	−0.55	0.586	0.527	0.053–5.273
High School	−2.201	1.382	−1.59	0.111	0.111	0.007–1.663	0.295	1.155	0.26	0.798	1.343	0.140–12.930	−0.353	1.134	−0.31	0.755	0.703	0.076–6.475
University	−2.164	1.388	−1.56	0.119	0.115	0.008–1.743	0.196	1.161	0.17	0.866	1.217	0.125–11.837	−0.631	1.142	−0.55	0.581	0.532	0.057–4.993
Postgraduate/Doctoral	−2.108	1.414	−1.49	0.136	0.121	0.008–1.941	0.286	1.194	0.24	0.810	1.332	0.128–13.822	−0.71	1.17	−0.61	0.544	0.491	0.050–4.874
Below 15 K	−0.544	0.387	−1.41	0.159	0.580	0.272–1.238	−0.677 ●	0.398	−1.70	0.089	0.508	0.233–1.109	−0.475	0.409	−1.16	0.246	0.622	0.279–1.387
Between 16 K and 24 K	0.165	0.295	0.56	0.576	1.179	0.661–2.103	0.09	0.294	0.31	0.759	1.094	0.616–1.946	0.302	0.291	1.04	0.3	1.352	0.764–2.393
Between 25 K and 49 K	0.18	0.269	0.67	0.503	1.197	0.707–2.028	−0.243	0.266	−0.91	0.361	0.784	0.466–1.321	−0.427 ●	0.266	−1.61	0.108	0.652	0.388–1.098
Between 50 K and 69 K	−0.091	0.373	−0.25	0.806	0.913	0.439–1.896	−0.727 *	0.366	−1.99	0.047	0.484	0.236–0.990	−0.286	0.341	−0.84	0.402	0.752	0.385–1.466
Above 70 K	−0.503	0.41	−1.23	0.220	0.605	0.271–1.351	−0.880 *	0.418	−2.11	0.035	0.415	0.183–0.941	−0.09	0.444	−0.2	0.839	0.914	0.383–2.179
PK	0.862 ***	0.245	3.52	0.000	2.368	1.465–3.828	0.985 ***	0.244	4.05	0.000	2.679	1.662–4.320	0.476 *	0.232	2.06	0.040	1.61	1.023–2.535
CEA	1.212 ***	0.162	7.5	0.000	3.362	2.449–4.615	1.178 ***	0.162	7.27	0.000	3.248	2.364–4.465	1.145 ***	0.159	7.18	0.000	3.142	2.299–4.294
AU	0.448 **	0.151	2.96	0.003	1.565	1.163–2.105	0.531 ***	0.148	3.59	0.000	1.701	1.272–2.272	0.493 ***	0.15	3.3	0.001	1.638	1.221–2.196
CB	−0.239 *	0.110	−2.16	0.031	0.788	0.634–0.978	−0.171	0.111	−1.55	0.122	0.843	0.678–1.047	−0.207 ●	0.112	−1.85	0.064	0.813	0.654–1.012
/cut 1	−4.737	1.430					−1.784	1.200					−1.566	1.178				
/cut 2	−3.475	1.420					−0.045	1.193					−0.043	1.174				
/cut 3	−1.428	1.413					1.802	1.198					1.723	1.181				
/cut 4	0.681	1.413					3.092	1.208					2.843	1.193				
	Laying hens						Broilers						Sheep/Goats					
Predictor	β	SE	z	*p*	Odds Ratio	95% CI for OR	β	SE	z	*p*	Odds Ratio	95% CI for OR	β	SE	z	*p*	Odds Ratio	95% CI for OR
Sex Female = 1	0.143	0.221	0.65	0.517	1.154	0.749–1.779	0.221	0.227	0.97	0.330	1.247	0.800–1.944	−0.588 **	0.226	−2.60	0.009	0.556	0.357–0.865
25–34	0.388	0.324	1.20	0.231	1.474	0.782–2.779	0.15	0.335	0.45	0.656	1.162	0.602–2.240	0.648 ●	0.341	1.90	0.057	1.912	0.980–3.731
35–49	0.634 *	0.301	2.11	0.035	1.885	1.045–3.400	0.735 *	0.315	2.33	0.02	2.085	1.124–3.868	0.241	0.306	0.79	0.431	1.273	0.698–2.316
50–64	0.763 **	0.287	2.66	0.008	2.145	1.223–3.764	0.956 ***	0.297	3.22	0.001	2.601	1.454–4.654	0.429	0.284	1.51	0.131	1.536	0.880–2.677
over 64	−0.072	0.431	−0.17	0.868	0.931	0.400–2.167	0.093	0.476	0.2	0.844	1.098	0.432–2.788	0.34	0.45	0.76	0.45	1.406	0.582–3.390
Middle School	−2.470 ●	1.420	−1.74	0.082	0.085	0.005–1.368	−2.041	1.556	−1.31	0.190	0.130	0.006–2.741	0.503	1.187	0.42	0.672	1.654	0.162–16.949
High School	−2.485 ●	1.39	−1.79	0.074	0.083	0.005–1.270	−2.017	1.524	−1.32	0.186	0.133	0.007–2.639	−0.238	1.156	−0.21	0.837	0.789	0.082–7.601
University	−2.938 *	1.396	−2.1	0.035	0.053	0.003–0.818	−2.226	1.530	−1.45	0.146	0.108	0.005–2.166	−0.33	1.163	−0.28	0.777	0.719	0.073–7.025
Postgraduate/Doctoral	−2.558 ●	1.415	−1.81	0.071	0.078	0.005–1.241	−2.242	1.551	−1.45	0.148	0.106	0.005–2.218	−0.191	1.19	−0.16	0.873	0.826	0.080–8.514
Below 15 K	−0.534	0.389	−1.37	0.169	0.586	0.274–1.256	−0.268	0.404	−0.66	0.506	0.765	0.347–1.687	−0.519	0.4	−1.30	0.194	0.595	0.272–1.304
Between 16 K and 24 K	0.023	0.294	0.08	0.936	1.024	0.576–1.822	0.264	0.292	0.91	0.364	1.302	0.736–2.308	0.105	0.286	0.37	0.714	1.111	0.634–1.945
Between 25 K and 49 K	−0.171	0.262	−0.66	0.512	0.843	0.504–1.406	−0.153	0.266	−0.57	0.566	0.858	0.509–1.447	−0.202	0.264	−0.77	0.444	0.817	0.487–1.370
Between 50 K and 69 K	−0.429	0.349	−1.23	0.219	0.651	0.329–1.290	−0.191	0.354	−0.54	0.59	0.826	0.413–1.655	0.18	0.374	0.48	0.631	1.197	0.575–2.493
Above 70 K	−0.169	0.429	−0.39	0.694	0.845	0.364–1.958	−0.502	0.476	−1.05	0.292	0.606	0.239–1.539	0.086	0.421	0.20	0.838	1.09	0.478–2.489
PK	0.227	0.224	1.02	0.310	1.255	0.810–1.946	0.193	0.235	0.82	0.412	1.213	0.765–1.923	0.458 ●	0.237	1.94	0.053	1.58	0.994–2.513
CEA	1.029 ***	0.154	6.68	0.000	2.799	2.069–3.784	1.124 ***	0.162	6.93	0.000	3.078	2.239–4.226	0.981 ***	0.161	6.11	0.000	2.668	1.948–3.654
AU	0.407 **	0.145	2.81	0.005	1.502	1.131–2.001	0.392 **	0.150	2.6	0.009	1.480	1.102–1.988	0.313 *	0.148	2.12	0.034	1.368	1.024–1.826
CB	−0.076	0.107	−0.71	0.478	0.927	0.752–1.143	−0.256 *	0.113	−2.27	0.023	0.775	0.621–0.967	−0.128	0.112	−1.14	0.252	0.880	0.707–1.096
/cut 1	−3.449	1.424					−2.115	1.555					−3.188	1.214				
/cut 2	−2.152	1.420					−0.773	1.553					−1.354	1.198				
/cut 3	−0.636	1.417					0.753	1.557					0.512	1.196				
/cut 4	0.589	1.420					1.632	1.557					2.131	1.204				

Note: Significance level 0.10, 0.05, 0.01, 0.00, indicated by ●, *, **, ***, respectively.

## Data Availability

We are unable to share the data at this time; however, we are open to reasonable requests from researchers, provided they comply with ethical, legal, or confidentiality requirements. Please contact the corresponding author for further inquiries.

## Appendix A

**Table A1:** Results of O-logit regression using dataset including people who follow a vegan diet.

	Perceived Well-Being					
	Dairy Cattle	Beef Cattle	Swine	Laying Hens	Broilers	Sheep/Goats
	ß (SE)	ß (SE)	ß (SE)	ß (SE)	ß (SE)	ß (SE)
Female	−0.401 ● (0.229)	−0.344 (0.223)	−0.038 (0.224)	0.146 (0.221)	0.222 (0.227)	−0.584 ** (0.226)
25–34	0.334 (0.337)	0.306 (0.327)	0.445 (0.326)	0.406 (0.323)	0.16 (0.335)	0.683 (0.34)
35–49	0.315 (0.311)	0.552 ● (0.304)	0.429 (0.299)	0.633 * (0.299)	0.733 * (0.314)	0.3 (0.304)
50–64	0.325 (0.283)	0.882 ** (0.285)	0.484 (0.284)	0.782 (0.286)	0.968 ** (0.296)	0.462 (0.283)
over 64	0.533 (0.446)	0.397 (0.42)	−0.04 (0.444)	−0.055 (0.431)	0.104 (0.475)	0.372 (0.449)
Middle School	−1.661 (1.412)	0.46 (1.193)	−0.628 (1.174)	−2.458 (1.417)	−2.035 (1.553)	0.527 (1.187)
High School	−2.197 (1.385)	0.298 (1.157)	−0.352 (1.133)	−2.485 (1.387)	−2.018 (1.522)	−0.232 (1.156)
University	−2.133 (1.391)	0.213 (1.162)	−0.634 (1.142)	−2.946 * (1.394)	−2.231 (1.527)	−0.296 (1.163)
Postgraduate/Doctoral	−2.098 (1.417)	0.292 (1.195)	−0.713 (1.17)	−2.564 (1.412)	−2.247 (1.548)	−0.18 (1.19)
Below 15 K	−0.626 (0.382)	−0.736 (0.393)	−0.497 (0.405)	−0.553 (0.385)	−0.279 (0.401)	−0.623 (0.395)
Between 15 K and 25 K	0.136 (0.294)	0.079 (0.293)	0.318 (0.291)	0.042 (0.293)	0.276 (0.291)	0.081 (0.285)
Between 25 K and 50 K	0.15 (0.268)	−0.258 (0.264)	−0.412 (0.265)	−0.153 (0.261)	−0.141 (0.266)	−0.231 (0.263)
Between 50 K and 70 K	−0.126 (0.372)	−0.746 * (0.365)	−0.275 (0.341)	−0.416 (0.348)	−0.183 (0.354)	0.143 (0.373)
Above 70 K	−0.533 (0.41)	−0.897 * (0.417)	−0.076 (0.443)	−0.152 (0.429)	−0.491 (0.476)	0.058 (0.421)
PK	0.860 *** (0.245)	0.986 *** (0.244)	0.479 * (0.232)	0.232 (0.224)	0.195 (0.236)	0.457 (0.237)
CEA	1.208 *** (0.161)	1.181 *** (0.162)	1.156 *** (0.159)	1.040 *** (0.154)	1.133 *** (0.162)	0.977 * (0.16)
AU	0.446 ** (0.151)	0.532 *** (0.148)	0.498 *** (0.15)	0.412 ** (0.145)	0.395 ** (0.15)	0.313 * (0.148)
CB	−0.237 * (0.11)	−0.173 (0.111)	−0.215 (0.112)	−0.084 (0.107)	−0.262 * (0.113)	−0.128 (0.111)
/cut1	−4.713 (1.432)	−1.776 (1.201)	−1.54 (1.177)	−3.421 (1.421)	−2.099 (1.552)	−3.145 (1.213)
/cut2	−3.464 (1.423)	−0.026 (1.194)	−0.019 (1.173)	−2.126 (1.417)	−0.756 (1.55)	−1.332 (1.197)
/cut3	−1.409 (1.415)	1.82 (1.199)	1.751 (1.181)	−0.607 (1.415)	0.772 (1.554)	0.54 (1.195)
/cut4	0.699 (1.416)	3.111 (1.209)	2.872 (1.192)	0.618 (1.417)	1.651 (1.555)	2.159 (1.204)
Observations (n°)	393 (393)	393 (393)	393 (393)	393 (393)	393 (393)	393 (393)

Note: Significance level 0.10, 0.05, 0.01, 0.00, indicated by ●, *, **, ***, respectively.

**Table A2:** Tukey’s HSD test results showing the specific group means of the animal categories that are significantly different from each other.

Group 1	Group 2	Mean Difference
Beef cattle	Broilers	−0.68 ***
Beef cattle	Dairy cattle	0.34 ***
Beef cattle	Laying hens	−0.33 ***
Beef cattle	Sheep and Goats	0.31 ***
Beef cattle	Swine	−0.4 ***
Broilers	Dairy cattle	1.02 ***
Broilers	Laying hens	0.35 ***
Broilers	Sheep and Goats	0.99 ***
Broilers	Swine	0.28 ***
Dairy cattle	Laying hens	−0.67 ***
Dairy cattle	Sheep and Goats	−0.03 ***
Dairy cattle	Swine	−0.74 ***
Laying hens	Sheep and Goats	0.64 ***
Laying hens	Swine	−0.07 ***
Sheep and Goats	Swine	−0.71 ***

Note: Significance level 0.00, indicated by ***.
